# Decoding autophagy in neuro-tumor crosstalk: from underlying mechanisms to translational opportunities

**DOI:** 10.7150/thno.134896

**Published:** 2026-07-13

**Authors:** Huifang Cheng, Hongyao Li, Peiyan Liu, Xin Jin, Yi Qu, Bo Liu, Aizhuo Li

**Affiliations:** 1 Department of Ultrasound, The First Hospital of China Medical University, Shenyang, 110001, China.; 2 Department of Biotherapy, Cancer Center and State Key Laboratory of Biotherapy, West China Hospital, Sichuan University, Chengdu, 610041, China.; 3 Department of Obstetrics, The First Hospital of China Medical University, Shenyang, 110001, China.; 4 Department of Hematology, The First Hospital of China Medical University, Shenyang, 110001, China.

**Keywords:** autophagy, cancer neuroscience, cancer therapy, neurotransmitter, neurotrophin

## Abstract

Recent advances in cancer neuroscience have established the nervous system as an active regulator of tumor initiation and progression, emphasizing the complex bidirectional crosstalk between neural networks and oncology. In order to deconstruct the relationship between primary central nervous system malignancies and brain metastasis, this review systematically studies the specific mechanisms of neuron tumor synaptic communication, paracrine signaling, and neuroimmune interactions. Innovatively, autophagy is considered as a regulatory bridge connecting neuronal activity and tumor behavior. When functioning in neurons, autophagic flux determines synaptic plasticity and regulates neurotransmitter turnover. Inside tumor cells, autophagy may lead to uncontrolled proliferation and help evade immune surveillance. Integrating these molecular observations establishes the neuro-autophagy-tumor axis, and interconnected nodes in this axis provide emerging therapeutic strategies that target neural signaling and autophagy. These insights highlight the importance of incorporating neurobiological context into cancer research and position autophagy as a promising target for disrupting neural regulation in cancer therapy.

## Introduction

**Figure 1:**
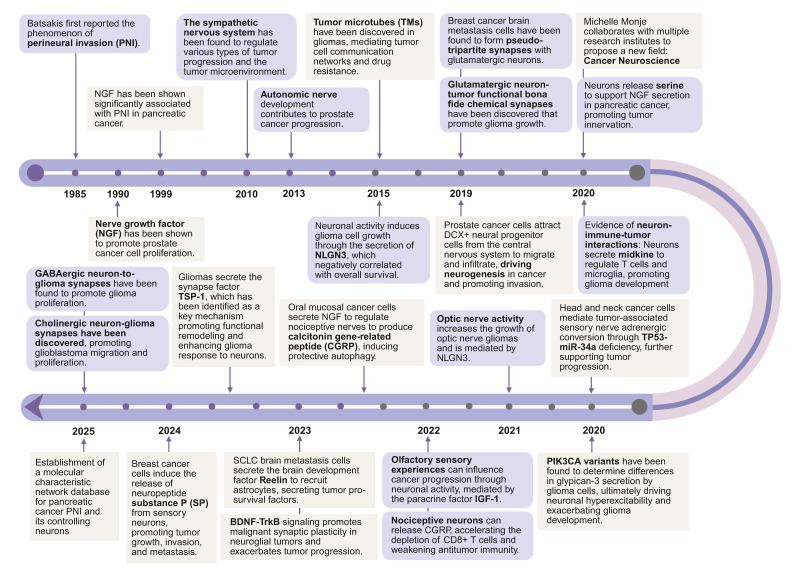
A brief history of cancer neuroscience. Key discoveries in cancer neuroscience have been summarized.

Epidemiological studies show an increase in the occurrence of tumors in the nervous system, particularly primary central nervous system (CNS) tumors such as glioblastoma and brain metastases 1. These tumors generally lead to a poor prognosis due to their aggressiveness, resistance to treatment, and the complexity of the neuroanatomical environment 2. Effectively, researchers are increasingly recognizing that chronic psychological stress, depression, and anxiety mediated by neuronal dysfunction may be risk factors that exacerbate tumorigenesis and progression 3. Similarly, growing evidence indicates the nervous system plays an active role in promoting tumor oncology. The concept of cancer neuroscience was proposed, and related fields have grown rapidly in recent years 4,5. One of the most striking findings is the discovery of neuron-to-tumor synaptic communication, wherein glioma cells form functional synapses with neurons 5. These synapses directly transmit excitatory electrical signals that enhance tumor cell proliferation and invasion. Similar phenomena have also been observed in brain metastases (**Figure 1**).

Neurons influence tumor progression through paracrine signaling. Neurons release neurotransmitters and neurotrophic factors. These factors bind to corresponding receptors expressed on tumor cells and activate oncogenic pathways. The nervous system also regulates immune responses within the tumor microenvironment (TME), contributing to immune escape and creating a niche favorable to tumor persistence 6. This neuro-immune-tumor axis is now recognized as a key driver of the failure of immunotherapies in neurological and systemic malignancies. Our current approaches to treating cancer might be missing a crucial aspect the role of the nervous system. Traditional therapies like chemotherapy and radiotherapy often cause neurotoxicity, namely chemotherapy-related cognitive impairment and radiation-induced brain injury (RIBI) 7,8. The nervous system may adapt to these treatments by activating pro-tumorigenic circuits 9,10. These observations indicate the crucial need to incorporate neural regulation into cancer therapy.

This intracellular degradation machinery strictly governs the complex physical interactions that link neural tissues with oncological lesions. When operating inside healthy neural networks, the autophagic flux dictates how synaptic junctions remodel themselves and actively calibrates the exact pool of available neurotransmitters, ultimately preventing the structural collapse of the neuron 11,12. Once hijacked by a malignant clone, this precise regulatory node forces the cell to navigate a biological fork: the degradative machinery either actively dismantles the tumor via programmed execution or metabolically shields the neoplasm to guarantee its survival 13.

Because it controls the rate at which presynaptic terminals release neurochemicals and how postsynaptic membranes recycle their receptors, this degradative network directly intercepts the cross-talk between nerves and cancer cells. This hijacked flux simultaneously dictates how invading macrophages polarize and how these immune sentinels present antigens when exposed to local inflammatory stress 14,15. High-resolution cellular profiling maps this dependency directly to invasive cellular architectures. Autophagic cascades physically assemble tumor microtubes (TMs) and tunneling nanotubes (TNTs), delicate membrane extensions that bridge distant cells and render the interconnected tumor network profoundly resistant to cytotoxic drugs 16. Utilizing these exact pathways, the expanding lesion actively recruits new neural fibers and physically tracks along existing perineural tracts (PNI), permanently forcing the surrounding neural architecture to supply the electrical inputs required for its own pathogenesis 17.

This review describes how the brain affects tumors. It looks at the main ways the nervous system influences both new brain tumors and those that have spread there. We also discuss the remodeling effects of tumors and their treatments on the nervous system. We summarize current drug strategies targeting the neuro-tumor axis. Importantly, this review extends beyond current cancer neuroscience frameworks by providing an in-depth exploration of autophagy as a critical regulatory bridge within these processes. We propose that autophagy acts as both mediator and effector in neural-tumor crosstalk, influencing tumor progression, immune evasion, and therapeutic outcomes. By integrating recent discoveries in neuroscience, tumor oncology, and autophagy research, this review provides a comprehensive framework for understanding the neuro-autophagy-tumor axis. It further outlines potential therapeutic opportunities, including the repositioning of neural signaling inhibitors, autophagy modulators, and dual-targeting agents to disrupt neuro-tumor interactions.

## 1. Physiological roles of autophagy in cellular and nervous system homeostasis

**Figure 2:**
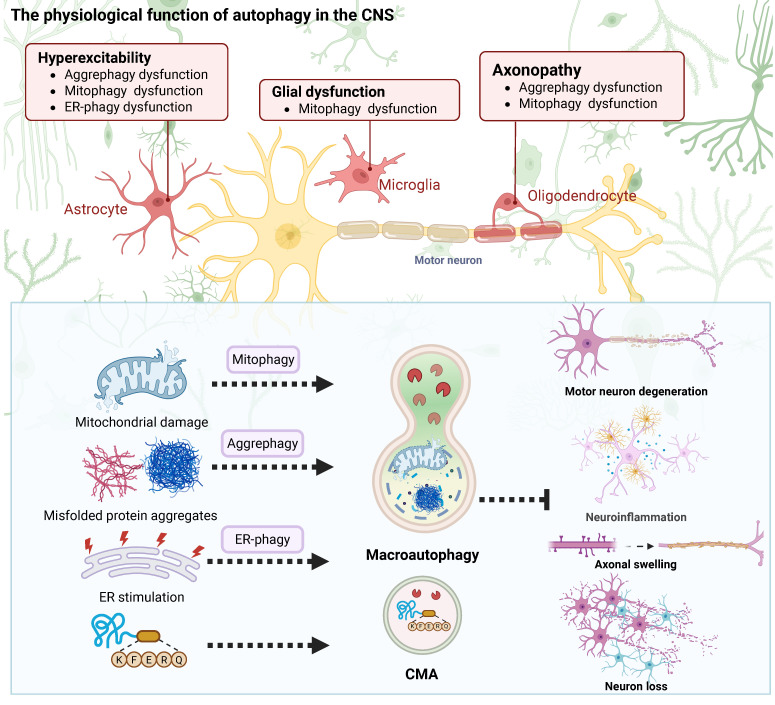
Physiological function of autophagy in the nervous system. Autophagy, including macroautophagy, chaperone-mediated autophagy, and microautophagy, plays essential roles in maintaining neuronal homeostasis. It supports organelle quality control, regulates synaptic development and plasticity, coordinates neuron–glia metabolic crosstalk, and buffers proteotoxic stress.

Autophagy is a conserved lysosome-dependent degradation pathway essential for cellular homeostasis, with particular importance in the nervous system. It comprises three major forms as macroautophagy, chaperone-mediated autophagy (CMA), and microautophagy, which collectively mediate the clearance of misfolded proteins, damaged organelles, and pathogenic aggregates. Neurons are in a postmitotic state and face a demand for metabolic resources that remains high across a long lifespan. These cells require autophagic flux to maintain the structural integrity of their networks (**Figure 2**).

Autophagy works as a recycling system, balancing biosynthesis and degradation in response to nutrients and stress. Macro-autophagy involves autophagosome formation and lysosomal degradation orchestrated by conserved ATG proteins. When specific components need to be cleared, receptors like Parkin and p62/SQSTM1 identify the targets. This process, called selective autophagy, then removes damaged mitochondria, parts of the endoplasmic reticulum, and clumps of proteins 18. CMA mediates HSC70-dependent lysosomal translocation of KFERQ motif-containing substrates via LAMP2A, whereas microautophagy contributes to basal turnover through direct lysosomal membrane invagination 19-21.

As neural tissue matures, its cleanup system does more than remove waste. It directly controls how neural networks survive and talk to each other. Neurons cannot divide again and need a lot of energy to work. For this reason, they completely depend on a constant, active cleanup process. This essential cleanup happens most importantly inside long axons and complex dendrites. There, it maintains healthy proteins and removes damaged cell parts. During brain development, this same cleanup process shapes the brain's ability to change and adapt. It removes unused synapses and manages the movement of neurotransmitter receptors and synaptic vesicles in established neural pathways. This activity affects learning, memory, and the overall stability of neural networks 22,23. Glial cells also use autophagy to help maintain balance in the nervous system. For example, astrocytes rely on it to clear away excess proteins and waste from outside cells. Microglia need autophagy to control inflammatory signals and prevent excessive brain inflammation. Oligodendrocytes require this process to maintain the protective myelin sheath and manage their membrane proteins 24. Autophagy often links to several brain disorders. Problems with this process are seen in conditions that involve poor synaptic function and abnormal myelin, including some neurodevelopmental and psychiatric illnesses 25,26. Impaired autophagy, which often occurs with aging, is a common feature in major neurodegenerative diseases 27,28. Its roles in synaptic plasticity, glial-neuronal crosstalk, and circadian coordination underscore the pathway’s complexity and therapeutic potential 29.

## 2. Autophagy in tumor biology

### 2.1 Autophagy in tumors of the nervous system

In primary nervous system tumors, autophagy exerts context-dependent dual functions. In glioblastoma and medulloblastoma, enhanced autophagic flux can sustain stemness, tumorigenicity, radio- or chemoresistance, and immune evasion, partly through pathways such as MST4-ATG4B signaling, AMBRA1-STAT3 activation, and autophagy-associated macrophage polarization. Conversely, under specific molecular contexts, autophagy may suppress tumor growth as shown in SPARC-overexpressing medulloblastoma, dopamine receptor D5 (DRD5)-activated pituitary adenoma, and CaMKII–Beclin1-regulated neuroblastoma. Thus, autophagy in primary nervous system tumors should be viewed as a dynamic and tumor-specific regulatory process, providing opportunities either to inhibit cytoprotective autophagy or to exploit pro-death autophagy for therapeutic benefit.

### 2.1.1 Glioblastoma

**Figure 3:**
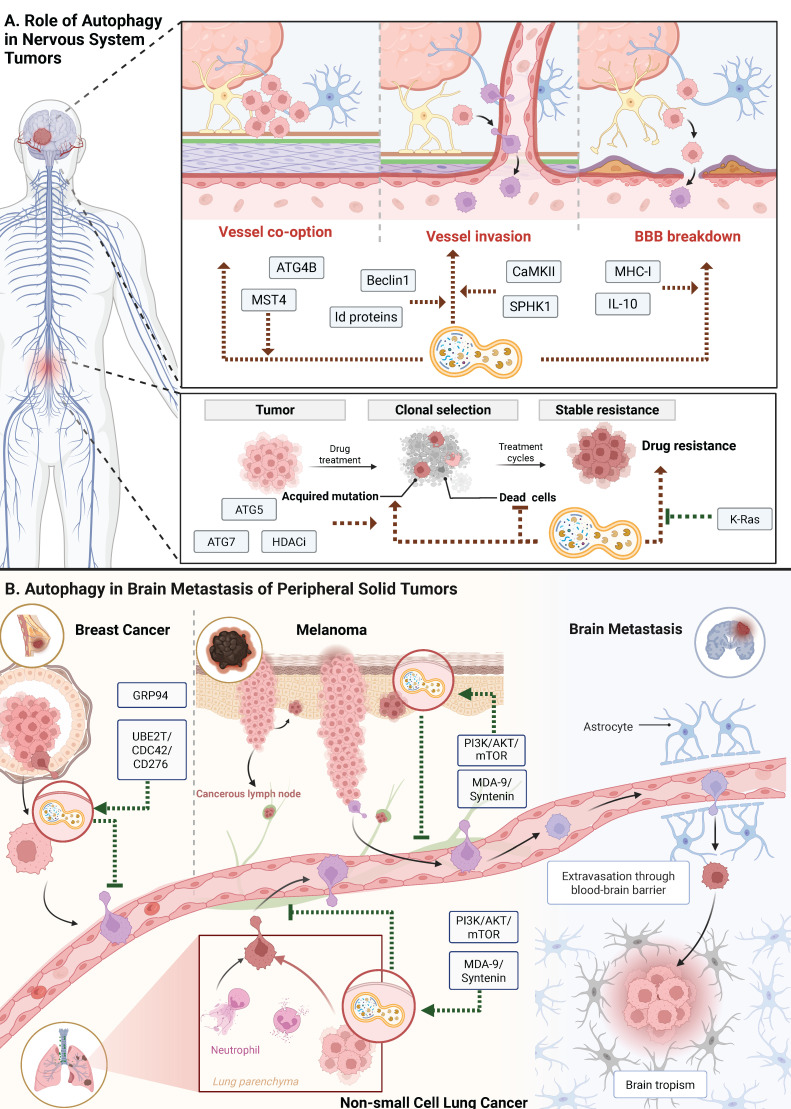
Pathogenic mechanism of autophagy in primary tumors and brain metastases. A. Role of Autophagy in Nervous System Tumors. Autophagy plays a key role in the occurrence and progression of central nervous system tumors, involving processes such as co-option of blood vessels, vessel invasion, and disruption of the blood-brain barrier. In addition, autophagy helps tumor cells acquire drug tolerance by regulating clonal selection and cellular stress response. B. Autophagy in Brain Metastasis of Peripheral Solid Tumors. Autophagy supports brain metastasis of peripheral solid tumors such as breast cancer, melanoma, and lung cancer by promoting tumor cell extravasation across the BBB and survival within the brain microenvironment. It modulates multiple signaling pathways that enable metastatic cells to adapt to the neural niche.

Autophagy plays dual roles in glioblastoma progression and therapy resistance through tumor-intrinsic survival pathways and immune microenvironment remodeling 30,31. Specifically, the kinase MST4 phosphorylates ATG4B at Ser383, thereby amplifying autophagic flux. This MST4-ATG4B phosphorylation axis sustains the stemness of glioblastoma cells and protects them from radiation 32. Simultaneously, glioblastoma-derived autophagy promotes an immunosuppressive microenvironment by releasing metabolites and cytokines that drive macrophage M2 polarization. This phenotypic shift, which impairs T-cell infiltration and downregulates the expression of MHC-I, allows the tumor to evade immune surveillance 33 (**Figure 3A**).

### 2.1.2 Medulloblastoma

In SPARC-overexpressing medulloblastoma cells, enhanced autophagic flux promotes lysosomal membrane permeabilization and caspase-dependent apoptosis, sensitizing tumor cells to stress-induced death 34. In contrast, the autophagy regulator AMBRA1 sustains cancer stem cells by coordinating pro-survival autophagy with STAT3 signaling, thereby promoting self-renewal and chemoresistance 35,36. Subtype-specific strategies that either exploit the apoptosis driven by SPARC or block the survival pathways supported by AMBRA1 may enhance therapeutic efficacy.

### 2.1.3 Pituitary adenoma

For pituitary adenomas, autophagy exerts context-dependent effects governed by mTOR signaling and dopamine receptor activation. When these tumors lack the protein DEPTOR, mTORC1 hyperactivates and suppresses autophagy. This suppression drives uncontrolled proliferation and causes resistance to dopamine agonists. Restoring DEPTOR expression inhibits mTORC1 and induces cytoprotective autophagy, resulting in cell cycle arrest and enhanced drug sensitivity 37. When DRD5 is activated, it induces too much autophagy through the AMPK and mTOR pathways, thereby inducing autophagic cell death through lysosomal membrane permeabilization and cathepsin-dependent caspase activation. This destructive form of autophagy seems to target only tumor cells, leaving normal pituitary cells unharmed 38. These results show that adjusting autophagy levels can be a way to treat cancer. Controlling this process can either stop tumors from growing or trigger their death, which supports using combination therapies that take advantage of these two different effects 39,40.

### 2.1.4 Neuroblastoma

Problems in autophagy genes like LC3B2 and ATG4A can disrupt the fusion of autophagosomes with lysosomes. This damage may make cells less able to handle stress from cancer-causing viruses. The resulting weakness promotes genetic instability and speeds up tumor growth 41. The kinase CaMKII adds a phosphate group to the protein Beclin1. This specific change significantly increases the cell's baseline level of autophagy. The boosted autophagy system then captures Id proteins and breaks them down. Removing these Id proteins disrupts the MYCN signaling pathway. As a result, the aggressive neuroblastoma cells stop dividing and change into mature, specialized neurons 42. In differentiated SH-SY5Y cells, glycolysis sustains autophagy induced by 4-hydroxynonenal, buffering oxidative stress and limiting apoptosis. But if researchers restrict this glucose metabolism, the autophagic defense collapses and shifts cells toward caspase-dependent death 43. Additionally, sphingolipid signaling exhibits marked cell-type specificity. While sphingosine kinase 1 promotes autophagic clearance of damaged organelles in neurons, it sustains cytoprotective autophagy by suppressing ceramide accumulation and apoptotic signaling in neuroblastoma cells 44.

### 2.2 Autophagy in brain metastasis of solid tumors

When solid tumors metastasize to the brain, disseminating cells activate autophagy to survive the severe metabolic and oxidative stresses that colonizing this new niche entails. By conferring resistance to anoikis and nutrient deprivation, autophagic flux helps tumor cells cross the blood-brain barrier (BBB). Following brain entry, the metastatic cells rely on autophagy to control mitochondrial quality and maintain metabolic flexibility, thereby promoting long-term adaptation to the nutrient-limited cerebral microenvironment. These conserved mechanisms render autophagy a targetable vulnerability for brain metastatic tumors (**Figure 3B**).

#### 2.2.1 NSCLC

Autophagy plays a context-dependent role in the formation and progression of brain metastases from non-small cell lung cancer (NSCLC), functioning both as an adaptive survival mechanism and a therapeutic vulnerability. Disruption of mitochondrial metabolism using mitochondria-targeted lonidamine inhibits electron transport chain complex I, leading to ATP depletion and activation of the AMPK/mTOR axis. This metabolic stress induces excessive mitophagy, which paradoxically results in mitochondrial loss and energy collapse, thereby impairing NSCLC cell survival and metastatic colonization in the brain 45. In contrast, genetic evidence from patients carrying the *ATG16L1* Thr300Ala polymorphism indicates that reduced autophagic capacity is associated with a lower incidence of brain metastasis. Impaired autophagy limits the ability of circulating tumor cells to withstand oxidative and metabolic stress during dissemination and reduces secretion of pro-metastatic factors, including matrix metalloproteinases, thereby restricting BBB penetration 46. Collectively, these studies indicate that autophagy supports metabolic adaptation during NSCLC brain metastasis, and that pharmacological or genetic inhibition of autophagic pathways may provide a potential strategy to limit metastatic progression.

#### 2.2.2 Breast cancer

Activation of lysosomal autophagy through the UBE2T/CDC42/CD276 axis enhances matrix metalloproteinase secretion, facilitating degradation of BBB components and enabling tumor cell extravasation 47. Additionally, molecular CMA, regulated by proteins such as GRP94, contributes to the stabilization of Snail transcription factors, promoting epithelial-mesenchymal transition and enhancing metastatic potential and resistance to microenvironmental stress conditions, including hypoxia and therapeutic interventions 48. In parallel, CMA, regulated by GRP94, stabilizes the transcription factor Snail, thereby promoting epithelial-mesenchymal transition and enhancing metastatic potential as well as resistance to hypoxia and therapeutic stress 49. The resident astrocytes can secrete KISS1 to activate autophagy in metastatic breast cancer cells, supporting metabolic flexibility and survival. Drug-based strategies can block cancer spread. Pharmacologists can use drugs that completely block lysosomes. They can also develop inhibitors that target specific proteins like CD276, GRP94, or the specific CMA recycling pathway 50,51.

#### 2.2.3 Melanoma

To colonize the brain, metastatic melanoma cells activate autophagy. This process helps them resist cell death and cope with the brain’s oxidative environment. The switch is controlled by MDA-9/Syntenin, which stabilizes the pro-survival PI3K/AKT/mTOR pathway 52. The tumor’s need for continuous autophagy creates a therapeutic opportunity. The compound trifluoperazine exploits this by blocking the fusion of autophagosomes with lysosomes. Autophagosomes accumulate, trapping waste and crippling the cell’s metabolism. This effect is selective, killing the melanoma cells without harming normal brain cells 53.

## 3. Neurological influences on tumors

**Figure 4:**
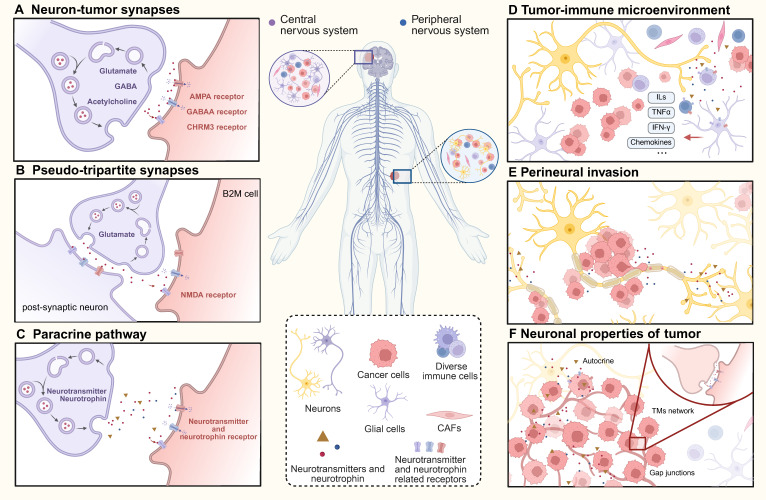
Neural inputs drive tumor progression. A. Neurons and tumor cells form real synaptic structures and regulate tumor progression through neurotransmitters such as glutamate, GABA, and acetylcholine. B. Tumor cells recruit at neuronal synaptic structures and form pseudo-tripartite synapses to receive neurotransmitter regulation. C. Neurons secrete factors into the tumor microenvironment, modulating tumor cell activity through paracrine signaling. D. Neuronal activity induces alterations in immune cells and remodels the immune microenvironment, impairing tumor immune responses. E. Peripheral neurons secrete factors that drive tumor cells growing along nerves and provide more accessible metastasis pathways. F. Tumor cells utilize TMs to form communication networks and accelerate progression via autocrine neurotransmitters and neurotrophins.

The nervous system is mainly made of neurons and glial cells. In the tumor microenvironment, these glial cells are important. They talk directly to cancer cells. Chronic stress, anxiety, and depression are known to be risk factors for cancer getting worse. Nervous system activity is associated with carcinogenesis and can significantly influence tumor development and metastasis (**Figure 4**) (**Table 1**). Autophagy acts as the exact molecular bridge linking neuronal firing to tumor expansion.

### 3.1 Direct neuron interaction

#### 3.1.1 Neuron-to brain tumor synaptic communication

Tumors located within the central nervous system are continually bathed in neural activity. This interaction occurs through a process termed neuron-to-brain tumor synaptic communication, which involves both direct and indirect forms of synaptic signaling. Gliomas are the most common malignant primary brain tumors in adults, originating primarily from neural precursor cells (NPCs) or oligodendrocyte precursor cells (OPCs) 54,55. Healthy OPCs form synapses with neurons. This natural interaction helps control their own development 56.

Glioma cells co-opt this same biological mechanism. They grow very thin, synapse-like structures called TMs. Using these microtubes, the cancer cells spread through healthy brain tissue. The microtubes also link glioma cells into a connected network. This network makes the tumor highly resistant to radiotherapy 57,58. To maintain these invasive structures, glioma cells must keep their internal skeleton stable. They also need to continuously move mitochondria to the growing tips of the microtubes. Both of these vital processes rely completely on well-functioning autophagy.

Glioma cells express various glutamate receptor subunits and genes associated with postsynaptic components 59. Approximately 10% of glioma cells form neuro-glioma synapses (NGSs), through which TMs establish functional, chemical synapses with neurons. These synapses generate unidirectional postsynaptic currents mediated by glutamatergic AMPA receptors, resulting in excitatory signals that depolarize glioma cells and enhance their proliferation 60. Additionally, neuronal activity induces non-synaptic, activity-dependent potassium currents. These currents are amplified via gap junction-mediated communication among glioma cells, forming an electrically coupled network that further supports tumor growth and malignancy 59. Cellular connectivity via gap junctions can be influenced by autophagy, which regulates the turnover of connexin proteins.

In addition to the well-characterized glutamatergic NGS, recent studies have identified functional GABAergic neuron-glioma synapses mediated by GABA receptors in diffuse midline gliomas (DMG). GABAergic input depolarizes DMG cells and promotes their proliferation, an effect that is further amplified by administration of the GABA-enhancing benzodiazepine lorazepam 61. Another study demonstrated that cholinergic neurons can also form cholinergic neuron-glioma synapses with glioblastoma cells, thus facilitating their migration and proliferation, and inhibition of CHRM3 receptors reduces tumor progression 62. Collectively, these findings suggest that glioma cells can hijack neuronal signaling, enhancing their survival and malignancy by increasing synaptic strength and synapse number. Such remodeling of neural circuits also contributes to cognitive impairment in affected individuals 63. At physiological synapse, autophagy directly controls how neurotransmitter receptors turn over and how vesicles recycle. When experimental models lack functional autophagic networks, the intracellular trafficking of GABA receptors completely stalls.

When cancer spreads from organs like the breast or lung to the brain, the new tumors also use the brain's own wiring to survive. Breast-to-brain metastases (B2BM) own a unique "pseudo-tripartite" architecture. Rather than constructing *de novo* synapses, the invading breast cancer cells position themselves around existing connections between healthy neurons. When these normal neurons fire, they release the neurotransmitter glutamate. The glutamate then activates NMDA receptors on the surface of the nearby cancer cells. This stolen signal drives the tumor cells to grow and invade 64. If the genes for these NMDA receptors are turned off, the cancer cells lose their ability to establish themselves in the brain tissue.

These metastatic cancer cells also develop features of GABA-releasing neurons. They make the same receptors and transporters that normal inhibitory brain cells use. This lets the cancer cells pick up extra survival signals from the surrounding brain network 65. SCLC in the brain uses a similar strategy. Researchers can use light to activate nearby brain neurons. When this happens, the neighboring cancer cells show an immediate electrical change. This shared electrical activity directly fuels the tumor's spread 66.

#### 3.1.2 Neuron-to extracerebral tumor synaptic-like communication

The PNS has also been implicated in cancer oncology. SCLC cells at the primary site look and act like nerve cells very early on and can generate electrical signals 67. These cells are believed to originate from neuroendocrine cells 68. These aggressive cancer cells can even grow long, thin extensions that look like axons, a process controlled by genes involved in nerve development. This lets them move more easily and spread to other parts of the body 69. Surgical interventions provide the ultimate proof of this anatomical dependency. Cutting the vagus nerve can stop the growth and spread of these lung tumors 66.

Besides these structures, cells can also connect through TNTs. These tiny tubes let cells share many things, from proteins and RNA to entire organelles, and are important in both health and disease 70,71. In the nervous system, TNTs facilitate communication between neurons and between neurons and glial cells, contributing to neurodevelopment and neurological disorders. In tumor cells, TNTs have been strongly associated with enhanced invasiveness and resistance to radiotherapy 72. Whether TNTs can serve as a communication bridge between neurons and tumor cells remains an important question for future investigation. Interestingly, the formation and function of TNTs are closely linked to autophagy-related processes, as they share molecular machinery for membrane trafficking and can transfer autophagosomes, potentially serving as a conduit for autophagic cargo between cells.

### 3.2 Paracrine neuro-tumor signaling pathways

#### 3.2.1 Paracrine secretion in the central nervous system

In the CNS, neuronal activity leads to the release of a variety of neurotransmitters and neurotrophins into the TME, which exert diverse regulatory effects on tumor cells. As previously described, tumor cells can directly receive neurotransmitters via synaptic connections, and they also express neurotransmitter receptors that respond to signals present in the surrounding environment. For instance, glutamatergic neurons can promote glioma proliferation and invasion not only through direct synaptic transmission but also via paracrine glutamate signaling 60. Similarly, cholinergic signaling through CHRM3 receptors and GABAergic signaling via GABA receptors have both been implicated in promoting tumor cell proliferation 62,65. Autophagic networks govern these exact physiological processes by dictating how neurons synthesize transmitters and recycle their vesicular pools.

The neuropeptide methionine enkephalin (MENK) can stop tumor growth. In gliomas, MENK binds to opioid receptors on the cancer cell surface. This binding causes the transcription factor NFAT1 to move into the nucleus, which then triggers cell death 73. This tumor-suppressive effect of MENK is also seen in cancers outside the brain, like melanoma and colon cancer 74. BDNF enhances the excitability and growth of glioma cells by increasing surface AMPA receptors. Genetically silencing or pharmacologically blocking the specific BDNF receptor TRKB inhibits this process 75. BDNF and GRP78 can also work together to stimulate tumor growth 75,76. Interestingly, autophagy helps regulate BDNF levels, and BDNF signaling can in turn affect autophagy, creating a two-way relationship.

Another neural factor, neuroligin-3 (NLGN3), also promotes cancer. When released, NLGN3 binds to glioma cells and over-activates the pro-growth PI3K-mTOR pathway 76. Furthermore, NLGN3 plays a crucial role in the establishment of NGSs 77. Its release from neurons is mediated by the sheddase ADAM10, and inhibition of ADAM10 or knockdown of NLGN3 significantly reduces glioma growth 78.

Not all paracrine interactions are limited to neuron-tumor communication. Within the TME of medulloblastoma, tumor granule neuron precursors can transdifferentiate into astrocyte-like cells, termed TuAstrocytes, which secrete interleukin-4 (IL-4) to activate microglia.

Once activated by the local microenvironment, these microglial sentinels release insulin-like growth factor 1 (IGF1). Rather than acting as a simple one-way signal, this specific secretion locks the immune cells and the malignant clones into a self-sustaining paracrine feedback loop, which continuously drives the expansion of the tumor 79. The cellular origin of IGF1 dictates its specific role in oncogenesis. In a different setting, neurons can also release IGF1 to start tumors. For instance, stimulating the sense of smell causes neurons to secrete IGF1, which can trigger glioma formation. This process relies on IGF1. When scientists reduce IGF1 levels in the brain's oligodendrocyte precursor cells, these cells stop multiplying, and tumor growth is blocked 80. Tumor-associated astrocytes fuel tumor growth. These astrocytes, reprogrammed by the TME and often found at the tumor edge, work by secreting lipocalin-2 (LCN2) and activating the STAT3 signaling pathway 81. Inhibiting LCN2 expression or blocking STAT3 signaling suppresses tumor growth, particularly in the Sonic Hedgehog subtype of medulloblastoma.

In brain metastases from SCLC, tumor cells secrete Reelin, a developmental cue that recruits astrocytes to the metastatic niche. These recruited astrocytes then secrete neuronal survival factors such as SERPINE1, which enhance SCLC proliferation and survival 82.

#### 3.2.2 Paracrine secretion in the peripheral nervous system

Within the PNS, the autonomic nervous system regulates the function of internal organs through the sympathetic (adrenergic) and parasympathetic (cholinergic) divisions. The sympathetic nervous system utilizes neurotransmitters such as epinephrine, norepinephrine, and dopamine, whereas the parasympathetic nervous system primarily relies on acetylcholine.

Neurotransmitters released from peripheral neurons influence cancer oncology. β-adrenergic signaling, in particular, has been shown to promote tumor cell proliferation, invasion, migration, angiogenesis, and resistance to cell death. These effects are largely mediated by activation of the cAMP/PKA and MAPK signaling pathways 83. Recent studies have revealed that adrenergic activity also accelerates colorectal cancer (CRC) progression via ADRA2A/Gi-mediated activation of Yes-associated protein 84. Furthermore, both ablation of adrenergic nerves and knockdown of adrenergic receptors significantly suppress prostate cancer growth and metastasis 85. Similarly, blockade of β-adrenoceptors (β-AR) has been shown to inhibit metastasis in breast cancer 86.

Cholinergic signaling has also been implicated in cancer progression. In gastric cancer, vagotomy reduces the activity of Wnt and Notch signaling pathways, consistent with findings that cholinergic input enhances Wnt signaling and promotes tumorigenesis 87. Cholinergic pathways similarly contribute to prostate cancer metastasis. However, these effects are context-dependent; for example, in pancreatic cancer, cholinergic signaling appears to suppress tumor growth, suggesting that neuronal subtypes may exert cancer-specific effects depending on the tumor type 88.

Substantial evidence indicates that N-methyl-D-aspartate receptors (NMDARs) are expressed in cancer cells outside the CNS, responding to glutamate released via autocrine and paracrine signaling 89. In pancreatic ductal adenocarcinoma (PDAC), glutamate from neurons induces calcium influx via NMDARs, activating the Ca^2+^-dependent CaMKII/ERK-MAPK pathway and enhancing glycolysis to promote PNI 90. Additionally, cancer cells adapt to oxidative stress and support rapid growth by upregulating glutamine metabolism through glutamate/glutamine interconversion. Importantly, the abovementioned β-adrenergic, cholinergic, and calcium signaling pathways are directly associated with the induction of autophagy in various types of cancer, positioning autophagy as a key mediator of tumor progression mediated by these pathways.

The PNS also secretes a variety of neurotrophic factors. Beyond their roles in axonogenesis and neural recruitment in the CNS, neurotrophins play critical roles in tumor progression. PNI, the invasion of tumor cells into and along nerve fibers, is associated with increased tumor aggressiveness and poor prognosis. Nerve fibers serve as low-resistance conduits for migration, while neurotrophins and chemokines secreted by neurons act as chemoattractants that facilitate PNI. For instance, glial cell line-derived neurotrophic factor (GDNF), released by nerves, attracts cancer cells via activation of RET receptors. The RET co-receptor GFRα1, expressed by cancer cells and secreted by nerve cells, enhances this signaling. Even in the absence of GFRα1 expression in cancer cells, nerve-derived GFRα1 promotes PNI through GDNF-RET signaling 91. Similarly, artemin, another neurotrophic factor, contributes to the neural invasion and dissemination of pancreatic cancer cells 92. Schwann cells, key components of peripheral nerves, can also promote PNI by secreting NCAM1, facilitating tumor cell migration toward and into nerves 93.

NGF, which supports neuronal survival and differentiation, has been shown to induce tumor cells to migrate toward nerves and recruit neuronal elements into the TME 94. NGF promotes tumor cell survival, migration, and invasion, especially in pancreatic and gastric cancers, via TrkA receptor activation and downstream PI3K/AKT and MAPK signaling 95. Under nutrient deprivation, oral mucosal cancer cells secrete NGF, which stimulates nociceptors to release calcitonin gene-related peptide (CGRP). This peptide, in turn, enhances tumor cell growth 96. BDNF, similar to its role in gliomas, promotes tumor invasion and metastasis in breast and ovarian cancers through activation of TrkB-mediated signaling pathways 97. Interestingly, there is growing recognition of crosstalk between neurotransmitters and neurotrophic factors. β-AR activation enhances NGF secretion, axonogenesis, and tumor proliferation 98. β2-adrenergic nerves also stimulate cancer-associated fibroblasts (CAFs) to express NGF, thereby promoting sympathetic innervation and driving CRC progression 84. β-AR activation increases catecholamine secretion, and catecholamines have been shown to induce NGF production in PDAC cells 99,100. Additionally, neuro-related cholinergic factors can stimulate NGF expression and promote gastric cancer development 87.

Peripheral neurons also release serine, which triggers metabolic crosstalk and increases NGF expression and secretion in PDAC, further enhancing tumor innervation and growth 101. Furthermore, neurons release substance P (SP), a neuropeptide that indirectly supports breast cancer growth, invasion, and metastasis. SP acts on TACR1 to induce tumor cell death, releasing single-stranded RNAs that activate TLR7 in adjacent tumor cells, thereby driving metastatic gene expression 102.

Finally, nerve fibers contribute to PNI not only by providing structural pathways, but also by secreting matrix metalloproteinases (MMPs), fibronectin, vascular endothelial growth factor (VEGF), and other molecules that remodel the TME. PNI is also driven by tumor cell-derived factors, which will be discussed in the following section.

### 3.3 Neuro-immune regulation of the tumor microenvironment

In addition to the direct regulation of tumor cells by neuronal signals, immune cells constitute a critical component of the TME and secrete a variety of regulatory molecules that influence both tumor cells and neurons. Notably, there is significant crosstalk between the nervous and immune systems. Immune cells express receptors for various neurotransmitters, enabling them to be modulated by neuronal input. Conversely, immune cells can also synthesize and release neurotransmitters, acting in both paracrine and autocrine manners.

Norepinephrine is released into the tumor microenvironment and binds to specific receptors on immune cells. This interaction does not send a normal physiological signal. Instead, it suppresses the anti-tumor immune response. One key effect is that receptor activation increases the expression of the immune checkpoint protein PD-1. Concurrently, this signaling cascade, hijacking pathways normally reserved for acute stress responses, dictates the suppressive behavior of local macrophages and myeloid-derived suppressor cells (MDSCs). It reprograms T-cell metabolism, impairing their ability to activate effectively and sustain anti-tumor functions 103. When dopamine engages D1-like receptors, the resulting biochemical cascade activates effector T lineages and aggressively amplifies the cytotoxicity of NK cells. Activation of D2-like receptors produces the opposite effect, suppressing natural killer cell activity and concurrently restraining the expansion of regulatory T cells (Tregs) 104. The parasympathetic neurotransmitter acetylcholine, in contrast, primarily delivers anti-inflammatory signals that help modulate overall immune activity 105.

#### 3.3.1 Neuro-immune crosstalk in the central nervous system

Within low-grade gliomas (LGGs) driven by neurofibromatosis type 1, local firing neurons actively synthesize and release the growth factor midkine. Intercepting this neural signal, adjacent CD8⁺ T cells undergo immediate activation, an acquired state that forces the lymphocytes to secrete the chemokine CCL4. Notably, CCL5 expression is negatively correlated with patient survival in LGG, providing direct evidence for the existence of a neuron–immune–tumor regulatory axis 106,107.

In gliomas, pharmacological inhibition of dopamine receptor D2 modulates TAM immune metabolism, leading to a pro-inflammatory TME 108. Additionally, infusion of BDNF significantly reduces microglial/macrophage infiltration and CD68 expression, thereby inhibiting glioma migration 109. BDNF also stimulates IL-15 production in microglia, which enhances IFN-γ secretion by NK cells and reprograms myeloid cells toward an anti-tumor phenotype 110.

Astrocytes, another key component of the CNS TME, express β₂-ARs. Activation of β₂-ARs modulates TNF-α expression and its downstream inflammatory gene network, influencing inflammatory homeostasis in the CNS 111. Astrocytes facilitate glioma progression by secreting interleukin-6. This cytokine engages IL-6 receptors on tumor cells, triggering the STAT3 signaling pathway that drives proliferation and invasion 112.

In drug-resistant breast cancer brain metastases, elevated levels of the neuronal compound N-acetylaspartate (NAA) and its transporter NAT8L suppress the activity of key immune cells like NK and CD8⁺ T cells. Tumor cells appear to co-opt this native neuro-immune checkpoint to avoid elimination 113. NAA-secreting neurons may facilitate immune evasion in both metastatic and primary CNS tumors 114.

#### 3.3.2 Neuro-immune crosstalk in the peripheral nervous system

The immune composition of the PNS TME is diverse, primarily comprising TAMs, T cells, NK cells, MDSCs, and B cells. Previous studies have demonstrated that norepinephrine signaling, released upon activation of intestinal sympathetic neurons, enhances macrophage-mediated immune responses to bacterial infections, confirming neuro-immune communication between intestinal neurons and macrophages 115. Sensory neurons, excluding autonomic nerves, release the neuropeptide CGRP, which impairs anti-tumor immunity by promoting CD8⁺ T cell depletion 116.

Drug repurposing research has shown that propranolol, a non-selective β_1_- and β_2_-adrenergic receptor blocker, inhibits CRC progression, likely through its role in blocking β₂-AR-mediated T cell depletion. Combination therapy with propranolol and the chemotherapeutic agent irinotecan exerts synergistically enhanced anti-tumor effects in CRC models 117. Inhibition of β_2_-adrenergic stress signaling also improves the efficacy of radiotherapy 118.

Further studies have demonstrated that activation of the sympathetic nervous system enhances β₂-AR signaling in CD8^+^ T cells, impairing their anti-tumor activity and promoting tumor immune evasion. Pharmacological inhibition of β_2_-AR can augment the effectiveness of anti-PD-1 immunotherapy 119. Moreover, chronic stress-induced adrenergic signaling disrupts T cell metabolism by depleting glycolytic and oxidative phosphorylation capacity, leading to immunosuppression in melanoma models 120.

Sympathetically released catecholamines activate β-adrenergic receptors on MDSCs within the TME, promoting the expression of immunosuppressive molecules such as arginase-1 and PD-L1 via STAT3 signaling, and enhancing their capacity to suppress T cell proliferation 121. Additionally, β_2_-AR signaling reprograms MDSC metabolism by reducing glycolysis while increasing oxidative phosphorylation and fatty acid oxidation, thereby reinforcing immunosuppression 122. Epinephrine and norepinephrine have also been shown to inhibit NK cell cytotoxicity and cytokine production 104.

Psychological stress further modulates tumor immunity. Sympathetic release of norepinephrine induces prostate tumor cells to secrete neuropeptide Y via β_2_-AR signaling, which promotes MDSC recruitment and IL-6 secretion. IL-6 subsequently activates STAT3 signaling and facilitates tumor progression 123,124. Excessive activation of cholinergic pathways can inhibit CD8^+^ T cell infiltration and decrease the Th1/Th2 ratio, contributing to an immunosuppressive microenvironment 125. The secretion of GDNF by perineurial macrophages is a key step that facilitates pancreatic cancer invasion. This neurotrophic factor acts by activating RET signaling pathways in the tumor cells 126.

Neuronal signaling molecules can influence cancer pathophysiology independently of neuronal structures. In the tumor-immune microenvironment, NGF signaling via TrkA receptors impairs IFN-γ signaling in melanoma cells, reduces T and NK cell infiltration, and suppresses T cell function 127. Similarly, BDNF contributes to immunosuppressive TAM reprogramming in lung adenocarcinoma 128. In prostate cancer, activation of leukemia inhibitory factor receptor signaling upregulates BDNF, promoting neuroendocrine differentiation and creating an immunosuppressive TME 129.

Other neurotransmitters also exert immunomodulatory effects. For instance, GABA suppresses T cell responses and infiltration in CRC, promoting a TAM-mediated immunosuppressive phenotype. Serotonin influences CD8^+^ T cell infiltration and modulates immune function 5. MENK, a neuropeptide, acts as a crucial immunoregulatory bridge between the nervous and immune systems. In lung and colorectal cancers, MENK remodels the tumor immune microenvironment by increasing the infiltration of macrophages, NK cells, and CD4^+^ and CD8^+^ T cells, thereby countering immune escape and resistance 130,131. Furthermore, nociceptor neurons in sensory nerves, except autonomic nerves, release a neuropeptide called CGRP, which impairs anti-tumor immunity by accelerating the depletion of CD8^+^ T cells 116. It is worth noting that many of the immunomodulatory effects of these molecules on the TME are mediated by autophagy, such as increasing tumor immunogenicity through autophagy activation.

The latest research has also discovered that cancer cells under immune pressure stimulate transcription factor 4 and secrete slit guidance ligand 2, which later activate tumor-draining nociceptive neurons. This action not only exacerbates cancer-induced pain but also induces nociceptive neurons to secrete CGRP, contributing to the remodeling of tumor-draining lymph nodes into an immunosuppressive state 132.

Finally, the nervous system exerts systemic control over the immune response. It modulates immune cell trafficking through cholinergic, adrenergic, and sensory peptidergic signaling and possesses "immune memory" capabilities, whereby neurons reinitiate specific immune responses upon reactivation by prior immune stimuli.

### 3.4 Autophagy-related regulation of the neural-tumor axis

**Figure 5:**
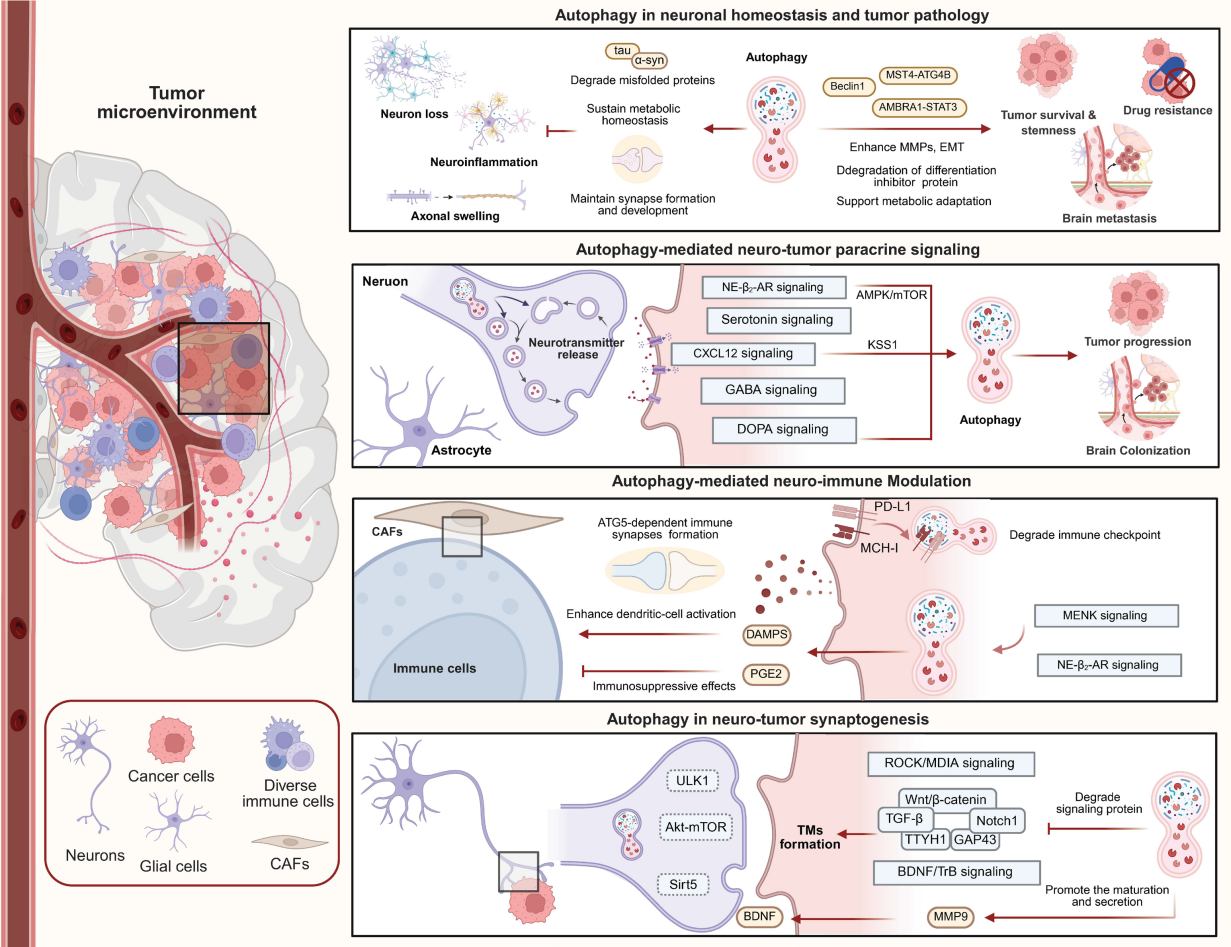
Autophagy maintains neuronal homeostasis while exerting context-dependent effects on tumor survival, stemness, metastasis, and therapy resistance. It also mediates neuro–tumor paracrine signaling by integrating neurotransmitter, neurotrophic, and stromal cues, and modulates immune responses through antigen presentation, immune-checkpoint regulation, DAMP release, and immunosuppressive mediators. The bottom panel illustrates a proposed mechanistic model in which autophagy may influence tumor microtube formation, synaptic plasticity, and neurotrophic signaling.

Given the central role of autophagy in neuronal and tumor biology, it has been proposed as a potential regulatory link in the neuro-tumor axis. Here, we discuss the potential mechanistic involvement of autophagy in paracrine neuro-tumor signaling pathways, neuro-immune regulation, and neural-tumor synapse formation (**Figure 5**). Based on these associations, the evidence supporting a role for autophagy in neuro-tumor interactions can be regarded as a proposed mechanism that deserves future experimental validation.

In terms of paracrine signaling and TME remodeling, autophagy plays a crucial role through interactions with neurotransmitters and neurotrophic factors. Autophagy participates in neurotransmitter synthesis and synaptic vesicle turnover. For example, autophagy maintains the homeostasis of tyrosine hydroxylase-positive neurons and supports dopamine synthesis 133, while autophagy induction in hepatocytes upregulates DOPA decarboxylase expression 134. In neurons, neurotransmitter release depends on synaptic vesicle recycling, and autophagy regulates the degradation and turnover of presynaptic membranes and vesicular proteins 135. Importantly, presynaptic autophagy is directly coupled to synaptic vesicle cycling through ATG9 trafficking 136. Activity-dependent exo-endocytosis of ATG9 links neuronal activity with autophagosome biogenesis at presynaptic terminals, providing strong mechanistic evidence that autophagy participates in synaptic communication machinery relevant to neuron-tumor signaling. Additional evidence supports a direct role for autophagy in neuronal secretory signaling. Glutamate-stimulated autophagosomes mediate the autophagic secretion of α-synuclein (α-Syn), and autophagy inducers increase α-Syn secretion 137. Since α-Syn expression is reduced in gliomas and inversely correlates with malignancy, restoration of α-Syn may exert tumor-suppressive effects 138. Together, these findings suggest that autophagy-dependent neuronal secretory pathways may influence glioma biology.

Most importantly, direct evidence also supports neurotransmitters and neurotrophic factors acting in autocrine or paracrine manners, regulating tumor cell autophagy. Chronic stress-induced activation of β2-adrenergic receptors promotes autophagy and accelerates gastric cancer progression in gastric cancer cells 139. Blockade of the DRD4 receptor inhibits the autophagy-lysosome pathway, inducing glioblastoma stem cells to undergo cell-cycle arrest and apoptosis 140. Serotonin exhibits context-dependent effects on autophagy. It can suppress starvation-induced autophagy in hepatocellular carcinoma and contribute to tumor progression through non-mTOR-dependent activation of mTOR downstream targets 141. Another study suggested that serotonin may promote hepatocarcinogenesis by inducing hepatic steatosis through activation of autophagy and the Notch pathway 142. Additionally, autophagy defects resulting from Atg7 deletion impair GABA receptor trafficking, highlighting a critical role for autophagy in neurotransmitter signaling 143. Furthermore, elevated serotonin signaling in depressive states activates the autophagy/P-STAT3 axis, creating an immunosuppressive microenvironment that promotes lung adenocarcinoma progression 144. Collectively, these studies directly link neurotransmitter signaling, autophagy regulation, immune modulation, and tumor progression.

Strong evidence additionally exists for autophagy-mediated glial–tumor interactions. Hypoxic gliomas secrete exosomes rich in miR-423-3p, which further enhance astrocytic autophagy and establish an immunosuppressive microenvironment favorable for glioma progression 145. Similarly, astrocytes secrete the chemokine CXCL12, which downregulates KISS1 expression in breast cancer brain metastasis cells. This KISS1 downregulation triggers the activation of ATG5/ATG7-dependent autophagy pathways, providing the necessary metabolic and structural signaling to drive the metastatic invasion and colonization of breast cancer in the brain parenchyma 49. This astrocyte-tumor-autophagy signaling loop represents one of the most mechanistically complete examples of autophagy-dependent neural stromal regulation in metastatic brain tumors.

Autophagy also contributes directly to immune synapse regulation and neuroimmune interactions within the TME. The immunosuppressive effects of β2-AR signaling are mediated by increased autophagy and activation of the arachidonic acid cycle, which ultimately enhances the release of the immunosuppressive mediator PGE2 122. In cutaneous squamous cell carcinoma, MENK activates autophagy and stimulates DAMP release, thereby enhancing dendritic-cell activation and tumor immunogenicity 146. Besides, autophagy directly dictates intrinsic immune cell function in the TME. Defective autophagy in microglia accelerates NLRP3 inflammasome activation, which in turn modulates inflammatory responses and immune cell function, significantly influencing glioma progression and brain metastasis 147-149. Furthermore, immune cells also form immune synapses with target cells. CAFs form immune synapses with Tregs in an ATG5-dependent manner, and ATG5 knockdown reduces Treg infiltration and tumor development 150. Inhibition of the autophagy protein Beclin-1 restores cytotoxic T lymphocyte-mediated tumor cell killing 151. These findings strongly support the concept that autophagy bridges neural, immune, and tumor signaling networks.

Evidence linking autophagy to PNI. Several autophagy-associated proteins have been identified as independent risk factors for PNI and poor prognosis in PDAC. Specifically, ubiquitin C has been identified as a key molecular link bridging the metabolic demands of PNI and autophagic flux, providing a foundation for future functional studies 152.

Auxiliary evidence supports the hypothesis that autophagy regulates TM formation, neural synaptic plasticity, and neurotrophic signaling. These conclusions are currently based primarily on inferences drawn from pathway interactions and indirect mechanisms, and warrant further validation. The synaptic ultrastructure of NGS is localized on TMs, and autophagy may be involved in TM formation. TM formation relies on dynamic remodeling of microtubules and actin, regulated by Rho GTPases, including RhoA, Cdc42, and Rac1 57. These factors activate ROCK and MDIA signaling to coordinate protrusion formation 153. Autophagy plays a central role in cytoskeletal remodeling and selectively degrades microtubule-associated and RhoA-associated proteins through autophagic degradation 154.

Furthermore, multiple signaling pathways that coregulate TMs are themselves modulated by autophagy. The TGF-β pathway and its downstream mediator TSP1 promote TMs communication through calcium signaling enhancement 155. Autophagy has been shown to modulate TGF-β activity in a context-dependent manner, either by promoting p62-dependent degradation of TGF-β or by enhancing TGF-β signaling through Smad7 degradation 156,157. TMs suppress Wnt signaling to promote neuronal degeneration, while simultaneously exploiting Wnt/β-catenin-driven JNK/MMP signaling to reinforce TM expansion, forming a positive feedback loop 158. Autophagy has been proven to negatively regulate Wnt/β-catenin signaling through β-catenin degradation and relocalization in glioblastoma cells 159. Autophagy can also degrade Notch1, a Wnt pathway activator whose downregulation promotes TM formation, whereas Notch1 upregulation is observed in TM-deficient oligodendrogliomas 160.

Several neuronal growth- and synapse-associated proteins further link autophagy to TM-dependent neuron-tumor interactions. Growth-associated protein 43 (GAP43) and tweety-homolog 1 (TTYH1), which mediate axon growth and synaptogenesis in neurons, are enriched in TMs. GAP43 drives TM-dependent tumor invasion, interconnection, proliferation, and radio-resistance, and its expression is regulated by autophagy-associated proteins, with autophagy inhibition reducing GAP43 levels 57. Moreover, TTYH1 is an endolysosomal membrane protein, promoting autophagy and mitochondrial quality control in glial cells. TTYH1 deficiency or autophagy defects impair mitochondrial turnover, increase oxidative stress, and alter cellular metabolism toward glycolysis, thereby impairing TMs' integrity and glioma progression 161-163. Neurotrophic signaling provides a functional link between autophagy and malignant synapse plasticity. BDNF levels are regulated by autophagic degradation, while secretory autophagy enhances extracellular BDNF maturation through stress-induced upregulation of MMP9 secretion 164,165. In glioblastoma, autophagy inhibition activates the NT-3-TrkC signaling and p38 MAPK phosphorylation, promoting tumor cell survival 166. Additionally, reciprocal involvement between autophagy and TrkB/BDNF signaling has been reported, where inhibition of one pathway activates the other, and dual inhibition produces greater antitumor effects than single-pathway targeting 167.

These findings support a mechanistic link between autophagy and TM-associated neural plasticity programs, which may be linked to functional neuron–tumor synapses and warrants further research to confirm.

Furthermore, to facilitate a broader discussion, some studies originally extrapolated from neuronal autophagy biology, neurodevelopmental systems, or non-neoplastic neurological disorders have also been proposed as hypotheses indicating that autophagy regulates neuro-tumor interactions. These hypotheses may be worth future validation within the context of neuro-tumor communication. For example, cylindromatosis promotes synapse elimination through Akt-mTOR-dependent autophagy activation, while mTOR inhibition rescues synaptic pruning defects 168. RPM-1 suppresses neuronal autophagy through regulation of UNC-51/ULK signaling, thereby affecting synapse maintenance and axonal development 169. Similarly, impaired neuronal macroautophagy in the prelimbic cortex contributes to synaptic dysfunction and anxiety-associated neural remodeling in chronic neuropathic pain models 170. SIRT5 regulates synaptic remodeling pathways and restores autophagic flux dysfunction in neurodegenerative models 171,172, while CHI3L1 influences TM connectivity and promotes autophagy through JNK signaling 173,174. These findings strongly support a role for autophagy in synaptic biology, but their extensions to neuron–tumor synapses remain speculative.

Overall, current evidence indicates that autophagy likely acts as a regulatory hub linking neuronal signaling, glial remodeling, neurotransmitter secretion, immune modulation, and tumor adaptation. The strongest support exists for autophagy-mediated neuroimmune signaling and glia–tumor communication, whereas the proposed role of autophagy in TM-dependent neuron–tumor synapse formation remains largely mechanistic and requires further experimental validation.

## 4. Tumor-induced remodeling of the nervous system

The connection between the nervous system and tumors is reciprocal. Tumor cells can directly invade neural tissue, secrete factors that disrupt neural function, and trigger systemic responses that profoundly alter both the structure and function of the nervous system.

### 4.1 Tumor-mediated neuromodulation and neural remodeling

First of all, primary brain tumors directly infiltrate brain tissue, causing compression or destruction of surrounding neurons and glial cells. The occupying effects of brain metastases can also disrupt the normal function of the nervous system. Beyond physical occupancy, neuron-to-brain tumor synaptic communication critically alters neuronal activity. For example, gliomas increase neuronal excitability, a hallmark characteristic of epilepsy. Seizures are commonly observed in glioma patients, primarily due to glioma cells secreting large amounts of the excitatory neurotransmitter glutamate, which hyperactivates AMPA and NMDA receptors on neuronal cells, leading to cell death 175,176. Additionally, gliomas can activate their own NMDAR and AMPAR in an autocrine manner, promoting glioma growth and invasiveness 177.

Elevated glutamate concentrations in the TME also strongly downregulate KCC2 levels. Peritumoral neurons experience a chloride imbalance due to deregulated expression of NKCC1 and KCC2, resulting in chloride efflux upon GABA receptor activation and generating a functional incentive 178,179. Paradoxically, endogenous GABAA receptor activity in glioma cells is associated with a reduction in their proliferation 180. Recent studies indicate that glioma cells with PIK3CA mutations differentially secrete glypican-3, contributing to neuronal hyperexcitability 181.

Tumor cells also induce neurogenesis and PNI by releasing paracrine factors, thereby creating a "neural microenvironment" that promotes tumor progression. In the PNS, pancreatic cancer cells use GDNF and ARTN to promote peripheral invasion and secrete NGF, which induces aberrant nerve fiber proliferation and extension into the tumor environment. This NGF-mediated axonogenesis is often associated with increased adrenergic or cholinergic signaling 100. Besides, tumor cells mediate neurogenesis in NPCs and facilitate neural reprogramming. Some studies have shown that peripheral tumors can establish communication with the brain at a distance. For instance, prostate cancer attracts DCX+ neural progenitor cells from the subventricular zone of the brain, prompting them to migrate through the bloodstream after crossing the BBB. These neural progenitor cells infiltrate the tumor, initiating intratumoral neurogenesis and generating new adrenergic neurons 182. Gliomas localized in functionally connected regions also secrete the synaptogenic factor thrombospondin-1 (TSP-1), which contributes to neuron-glioma interactions, ultimately remodelling neural circuits, promoting glioma progression, and impairing cognitive performance 63. Another study revealed that in head and neck cancer, loss of TP53 leads to loss of the microRNA miR-34a, contributing to tumor-associated axonogenesis of sensory nerves and adrenergic transformation, thereby supporting tumor progression 183.

Paraneoplastic syndromes are abnormal immune responses or ectopic hormone production induced by tumor products, including hormones secreted by tumors. When tumor cells express antigens similar to those found in neurons (e.g., Hu /Yo /Ri proteins), an autoimmune response is triggered, leading to antibody-mediated attack on the nervous system. This results in neurological disorders known as paraneoplastic neurological syndromes 184. Examples include opsoclonus-myoclonus ataxia syndrome in neuroblastoma, characterized by cerebellar and brainstem autoimmunity, and paraneoplastic cerebellar degeneration associated with SCLC.

Furthermore, tumor cells cause nerve cell dysfunction through abnormal metabolism, which leads to lactate accumulation and an enrichment of reactive oxygen species along with inflammatory mediators in the neural-TME 185. For example, CRC may cause sensory neuron dysfunction via cytokine and chemokine signaling, such as CXCL10 186. In addition to neuronal cells, breast and lung cancer cells can form a gap junction network with astrocytes by expressing PCDH7. These channels facilitate the transport of cGAMP from brain metastatic cancer cells, activate the STING pathway, and produce inflammatory factors like TNF and IFNα. This, in turn, activates the STAT1 and NF-κB pathways, contributing to tumor progression and chemoresistance 187.

### 4.2 Tumor therapy-induced neurotoxicity and neural dysfunction

Tumor treatment harms more than just the cancer. The radiation and chemotherapy used to fight the disease also damage the healthy nervous system. Many patients receiving these treatments develop problems with thinking and memory, a side effect often called "chemo-brain." This includes poor concentration, serious memory loss, and slower thinking. These drugs do not only target fast-growing cancer cells. They also attack and harm normal brain structure and function. Chemotherapy can hurt the brain by causing inflammation, increasing oxidative stress, and stopping the growth of new neurons 188,189. One common drug, paclitaxel, works by blocking cell division. However, this same action disrupts the microtubules that neurons need for their proper shape and function. This disruption leads to dysfunctional neuronal growth, development, and communication. Additionally, chemotherapeutic drugs like methotrexate, which inhibit DNA synthesis, can result in DNA damage accumulation and reduced generation of new neurons. Furthermore, treatments with adriamycin and methotrexate have been shown to reduce BDNF levels, thereby suppressing neurogenesis 190,191.

RIBI is another common complication of radiation therapy for tumors. This condition refers to brain tissue damage caused by radiotherapy, leading to neuronal and glial cell degeneration, necrosis, and CNS disorders. RIBI is primarily driven by direct DNA damage, apoptosis, vascular damage, and inflammation. For example, radiation prompts microglia to secrete pro-inflammatory chemokines, such as CCL2 and CCL8, which mediate CD8+ T-cell infiltration and contribute to chronic neuroinflammation 192. Conversely, some studies have indicated that radiotherapy may enhance neuronal-tumor connectivity by increasing neuronal activity 62.

### 4.3 Autophagy-related regulation of tumor-induced neural remodeling

Autophagy not only mediates the neurological impact on tumor progression, but also plays an indispensable role in how tumors affect the nervous system. In terms of tumor progression, autophagy has duality. On the one hand, autophagy supports tumor cell survival by removing defective organelles, reducing oxidative stress, preventing DNA damage, and resisting metabolic stress, thus promoting tumor cell survival. On the other hand, autophagy facilitates neurological invasion by influencing processes related to tumor metastasis. Additionally, autophagy contributes to the creation of a suppressive tumor immune microenvironment, which aids in immune evasion. In contrast, excessive autophagy activation can lead to cytotoxic effects, inducing cell death and inhibiting tumor progression 193. This dual role is likely dependent on factors such as tumor type, progression stage, and the TME.

Autophagy also mediates the suppressive immune microenvironment within tumors. It not only regulates the transcription and translation of immune checkpoint molecules such as PD-L1 but also facilitates the autophagic degradation of PD-L1 and MHC-I. This promotes immune evasion by tumor cells, influencing their ability to invade the nervous system 194. Thus, autophagy plays a critical role in both promoting tumor survival and invasiveness, further impacting the nervous system.

Beyond its role in intracellular degradation and recycling, autophagy also regulates extracellular release pathways 195. Autophagy influences the accumulation and release of inflammatory factors such as IL-6, CXCL8, ROS, and HMGB1, which can have either anti-inflammatory or pro-inflammatory effects 196. These released inflammatory factors may contribute to neuronal damage in the tumor’s vicinity. Moreover, autophagy is involved in neurotransmitter release in neurons, and tumor cell-derived neurotransmitter crosstalk and neurotrophic factors may also depend on autophagy for secretion. For instance, in pancreatic cancer, the NGF/ATG paracrine pathway activates autophagy in Schwann cells, which not only repairs nerves and promotes nerve fiber extension towards tumor cells but also drives tumor cells towards nerve-directed growth, facilitating neural infiltration 17. Blocking both NGF and autophagy inhibits PNI. In oral mucosa carcinoma, autophagy-induced neuronal growth inhibition is associated with CGRP-mediated protection, which activates autophagy in cancer cells by disrupting Rap1-mediated mTOR-Raptor interactions 96. Furthermore, tumor cells themselves exploit autophagic pathways to dictate the composition of the surrounding TME. For instance, glioblastoma utilizes secretory autophagy to release PAI-1 into the extracellular space, supporting an immunosuppressive niche. Pharmacological inhibition of this autophagic release restricts extracellular PAI-1, thereby remodeling the TME into a pro-inflammatory, anti-tumor state and significantly prolonging survival 197.

Neurotoxicity induced by tumor treatments, such as chemotherapy and radiotherapy, is also linked to autophagy. For example, mitoxantrone, a chemotherapeutic agent used to treat various tumors and multiple sclerosis, induces neurotoxicity through excessive autophagy, leading to neuronal death 198. Similarly, sunitinib, a targeted drug against VEGFR, causes cognitive and memory impairments by impairing autophagy in neurons 199.

RIBI is commonly associated with cerebrovascular dysfunction, which is considered a significant cause of cognitive impairment. Recent studies indicate that autophagy defects in pericytes, which are part of the neurovascular unit, promote BBB dysfunction by inducing senescence. This contributes to cognitive decline and aids in glioma cell growth and invasion 200. Interestingly, autophagy activation through rapamycin eliminates senescent cells and significantly alleviates radiation-induced cognitive dysfunction.

Furthermore, autophagy plays a crucial role in chemoresistance in gliomas. For example, temozolomide (TMZ) treatment induces autophagy and leads to treatment resistance, which is associated with impaired down-regulation of ATG9B expression by downregulated DAB2IP through the Wnt/β-catenin pathway in drug-resistant cells 201. Similarly, autophagy flux has been shown to correlate with glioma sensitivity to radiation, and blocking autophagy may enhance treatment efficacy 202. In conclusion, autophagy mediates resistance to conventional tumor therapies and indirectly contributes to the tumor's detrimental effects on neural tissue.

## 5. Therapeutic strategies for targeting neural-tumor interactions

With the rapid advancement of cancer neuroscience, an increasing number of neural–tumor interactions are being elucidated. These discoveries hold significant potential for translation into novel cancer therapies. Based on mechanistic insights into the neural–tumor interaction network, key signaling molecules, such as neurotransmitters, their receptors, and neurotrophic factors, have emerged as promising targets for the development of targeted therapeutics. Furthermore, these findings offer a rationale for the repurposing of existing drugs that modulate neural signaling pathways (**Table 2**).

### 5.1 Current therapeutic strategies targeting neural-tumor communication pathways

From the perspective of targeting neurotransmitters, inhibition of β-adrenergic receptor signaling has been shown to suppress both neuronal secretion and norepinephrine stimulation originating from various sources within the TME. This inhibition can impede tumor progression by reducing tumor cell proliferation and metastasis, modulating the immunosuppressive environment, and inducing tumor cell death 203. Representative agents include non-selective β-blockers such as propranolol and carvedilol. Propranolol, a lipophilic compound capable of crossing the blood–brain barrier, has demonstrated antitumor effects in preclinical studies across a range of cancers, including colorectal, prostate, breast, ovarian, and various hematologic malignancies 204. Both propranolol and carvedilol are currently being evaluated in clinical trials.

Inhibiting glutamate receptors offers a direct way to stop tumors driven by neural activity. AMPA and NMDA receptors help pass excitatory signals from neurons to cancer cells. Blocking them with drugs can halt cancer cell multiplication and also shield healthy neurons from harm. A known NMDA receptor blocker, memantine, is used in the clinic. It is given with radiotherapy to lessen brain injury and cognitive side effects. It has also shown immunomodulatory and antitumor effects in preclinical models 205. Talampanel, an AMPA receptor inhibitor, demonstrated safety and therapeutic potential in glioma patients when combined with radiotherapy and TMZ, suggesting its utility in glioblastoma treatment 206. Perampanel, another AMPA receptor antagonist, has been reported to reduce glioma invasion and progression by disrupting glutamatergic signaling between neurons and tumor cells 60. Additionally, it has shown efficacy in mitigating glioma-associated epilepsy and is currently under investigation in multiple clinical trials as an adjunct therapy.

Other neurotransmitter-targeting agents include the cholinergic receptor antagonist atropine, which has shown preclinical efficacy in breast and colorectal cancers by inhibiting epithelial–mesenchymal transition and alleviating immunosuppression 207,208. Bethanechol, a muscarinic receptor agonist, has also demonstrated effectiveness in PDAC and is under clinical evaluation 88.

ONC201 has been identified as an antagonist of dopamine receptors DRD2 and DRD3. It blocks AKT/ERK signaling in tumor cells and has antitumor activity in multiple cancer types like glioma, prostate cancer, and endometrial cancer by inhibiting proliferation, mediating apoptosis, and contributing to a pro-inflammatory microenvironment. ONC201 is being evaluated in a clinical trial in patients with advanced solid tumors and hematological malignancies 108,209. These neurotransmitter receptor antagonists are frequently incorporated into therapeutic strategies targeting nerve–tumor interactions, often through drug repurposing approaches.

Moreover, inhibition of neurotrophic factor signaling represents another crucial approach for disrupting the neural–tumor axis. Notably, targeting the NGF-Trk and BDNF-TrkB signaling pathways has shown therapeutic potential. CAFs, through interactions with sympathetic nerves, can promote tumor progression via NGF secretion within the TME. Inhibition of Trk signaling can disrupt these neuro-mesenchymal interactions and has been proposed as a strategy for CRC treatment 84. Tanezumab, a monoclonal antibody against NGF, has reached Phase III clinical trials for bone cancer, where it demonstrates efficacy in pain relief and inhibition of nerve infiltration (NCT02609828). Additionally, it has been reported to suppress neural invasion by pancreatic cancer cells and reduce nociceptive transmission 210. Pan-Trk inhibitors such as entrectinib and larotrectinib have been approved for solid tumors harboring NTRK gene fusions 211. These agents offer targeted therapeutic options for tumors driven by neurotrophic signaling and characterized by neural–tumor interactions.

Emerging targets such as the NLGN3-ADAM10 signaling axis also show promise. INCB7839, an ADAM10 inhibitor, is currently under clinical investigation for multiple brain and extracranial tumors, including glioma and breast cancer 77. Similarly, meclofenamate, an inhibitor of gap junctions, has been reported to disrupt TMs formation in gliomas, thereby impairing TM-mediated tumor cell communication 212.

Given the unique properties of the CNS, repurposing drugs capable of crossing the blood–brain barrier may offer an additional strategy to target the neural–tumor axis. Several antipsychotics and antidepressants have demonstrated antitumor activity 213. For example, aprepitant, a TACR1 antagonist approved for chemotherapy-induced nausea, has been shown to inhibit neuronal hyperactivation induced by SP released from breast cancer cells. This agent targets the neuropeptide/extracellular ssRNA-sensing axis and suppresses breast cancer growth and metastasis in various preclinical models 102.

Telmisartan, an angiotensin II type 1 receptor blocker with PPARγ agonist activity, has been shown to counteract the pro-tumorigenic effects of IL-6 secreted by astrocytes, suggesting its potential use as an adjuvant therapy in glioma patients, particularly those with hypertension 112. The FDA-approved drug gabapentin, used as an antiepileptic drug, can decrease glioblastoma proliferation through the inhibition of TSP-1 63. Likewise, rimegepant, an anti-migraine drug, has demonstrated efficacy in inhibiting oral mucosal carcinoma by blocking neurogenic CGRP signaling 96.

Additionally, the rabies virus, beyond its established use as a neural tracer, has been engineered to selectively induce apoptosis in glioma-connected neurons, thereby halting glioblastoma progression. This represents a novel viral strategy for glioblastoma treatment 62.

### 5.2 Therapeutic potential of autophagy-directed modulation of neural-tumor interactions

As previously discussed, autophagy serves as a critical mediator in the crosstalk between the nervous system and tumors: (1) plays an essential role in neuronal development, (2) regulates the release of neuron-related factors, (3) exerts a dual regulatory effect on tumor cells, and (4) functions as a downstream effector of neural–tumor interaction signaling. Therefore, targeting autophagy may disrupt neural–tumor interactions through multiple mechanisms, offering a novel avenue for cancer research and therapeutic intervention.

Evidence suggests that autophagy inhibition can impair neural–tumor dynamics. For instance, the autophagy inhibitor chloroquine suppresses autophagic flux in Schwann cells within the pancreatic cancer microenvironment, thereby interrupting NGF signaling derived from pancreatic cancer cell paracrine secretion. Simultaneously, it induces apoptosis in pancreatic cancer cells and prevents neural infiltration 17. Clinical pathological analysis has further revealed a positive correlation between PNI and elevated autophagy in pancreatic cancer patients 214. The antitumor effects of many neuro-modulatory drugs are mediated through changes in autophagy. Certain beta-blockers can inhibit autophagic flux, and this inhibition may be central to their anti-neurotropic activity 215. Conversely, memantine has been identified as an autophagy activator that restores autophagic flux and improves neuronal viability 216.

In addition, some preclinical evidence has shown that autophagy modulators combined with neuro-tumor axis blockers exhibit synergistic effects.

In CRC, BDNF inhibitor K252a, combined with chloroquine, has been shown to significantly reduce tumor cell metabolic activity and enhance apoptosis, demonstrating superior efficacy compared to monotherapies 167. Induction of autophagic dysfunction has also been reported to enhance the cytotoxic activity of propranolol against hemangioma cells 217. In addition, Chloroquine was shown to suppress ONC206-induced protective autophagy, thereby potentiating the pro-apoptotic effects of ONC206 and enhancing its antitumor efficacy in hepatocellular carcinoma 218. Similarly, chloroquine inhibited the protective autophagic response induced by imipramine in esophageal squamous cell carcinoma, further augmenting tumor suppression 219. Silencing of the autophagy-related gene ATG5 was likewise reported to enhance Melatonin-induced apoptosis in liver cancer cells 220.

Conversely, another study demonstrated that Larotrectinib induced autophagic cell death through activation of autophagic flux, whereas co-treatment with chloroquine disrupted its antitumor activity against CRC, highlighting the context-dependent and dual role of autophagy modulation in cancer therapy 221.

In addition, results from clinical trials of glioblastoma treatment using a combination of chloroquine and TMZ chemotherapy, along with radiation therapy, indicate that this combination regimen can prolong overall survival 222. However, further expanded clinical trials will require the development of more potent and less toxic autophagy inhibitors.

Interestingly, owing to their intrinsic ability to penetrate the blood–brain barrier, repurposed neuropsychiatric agents with antitumor activity, particularly antidepressants, have emerged as promising therapeutic candidates for brain malignancies. Notably, the antineoplastic effects of many of these agents appear to be closely associated with modulation of autophagy pathways. Among them, imipramine, maprotiline, fluoxetine, and escitalopram have been reported to induce autophagy, whereas nortriptyline, clomipramine, and paroxetine function predominantly as autophagy inhibitors. In contrast, sertraline and desipramine exhibit context-dependent effects on autophagy, acting either as autophagy inducers or inhibitors depending on the specific tumor setting 223.

Given autophagy’s inherently dual role, contextually promoting either tumor suppression or survival, its regulatory state within the neuro–neurosecretory factor-tumor axis is highly dependent on the spatial and temporal context of the TME.

In the context of neurodevelopment and neuronal homeostasis, physiological autophagy is primarily protective and functionally indispensable. Inducing autophagy or restoring autophagic flux is generally associated with maintaining neuronal stability and preventing neurodegeneration. Excessive inhibition of autophagy within the CNS may consequently impair neuronal survival, synaptic stability, and cognitive function. Conversely, during tumor progression and metastasis, autophagy frequently acts as an adaptive survival mechanism. Blocking this protective autophagy can thereby overcome microenvironmental adaptation and therapeutic resistance. In highly aggressive tumors such as glioblastoma and metastatic brain tumors, autophagy primarily functions as a pro-survival mechanism that supports tumor stemness, metabolic adaptation, immune evasion, and therapeutic resistance. For example, the MST4-ATG4B axis enhances autophagic flux to sustain glioblastoma stemness and radioresistance 32, while astrocyte-associated autophagy promotes brain metastasis adaptation to the cerebral microenvironment 49. Excessive or dysregulated autophagy can also induce tumor cell death and represents another exploitable therapeutic vulnerability.

Most importantly, in the currently strongest-supported neuro–tumor interaction models, autophagy primarily promotes tumor-supportive communication between neurons, glial cells, immune cells, and tumor cells, suggesting that selective inhibition of tumor-associated autophagy may provide therapeutic benefit. For instance, β2-adrenergic signaling promotes tumor progression through autophagy activation 139, while astrocyte-derived CXCL12 induces ATG5/ATG7-dependent autophagy to facilitate breast cancer brain metastasis 49. Similarly, hypoxic glioma-derived exosomes enhance astrocytic autophagy to establish an immunosuppressive microenvironment 145. In these contexts, autophagy inhibition may suppress neural-derived trophic support, reverse immunosuppression, and sensitize tumors to therapy. Consequently, in current therapeutic developments, pharmacological blockers of neuro-tumor interactions are increasingly being combined with autophagy inhibitors to achieve robust synergistic effects.

However, excessive systemic autophagy inhibition within the CNS may simultaneously impair neuronal synaptic homeostasis and disrupt normal neuroimmune regulation. Conversely, controlled autophagy activation in neurons or immune cells may preserve neuronal integrity and enhance antitumor immunity in selected contexts. For example, MENK-induced autophagy promotes DAMP release and dendritic-cell activation 146, while autophagy-mediated degradation of oncogenic signaling intermediates may suppress TM-associated plasticity and malignant synapse reinforcement. Thus, within the neural–tumor axis, the most promising future strategy may not involve global activation or inhibition of autophagy, but rather spatially and cell-type selective modulation, in which autophagy is inhibited in tumor-supportive compartments while preserved or restored in neurons and antitumor immune populations.

## 6. Concluding remarks and future directions

Cancer neuroscience reconstructs the traditional boundaries of oncology. The nerves in our body are closely linked to growing tumors. They do not just grow into TMEs, but also help tumors grow and resist treatment. Nerves talk to tumors through connections and by releasing special chemicals. Neurotransmitters and neurotrophic factors operating through β-adrenergic, glutamatergic, and cholinergic cascades form the molecular basis of these regulations. Several pharmacological agents have exhibited either preclinical or clinical evidence of efficacy in modulating neural inputs to tumors. Furthermore, repurposed CNS medications, such as antidepressants, antipsychotics, and anti-migraine agents, have demonstrated potential in disrupting neurogenic signaling within the TME.

Autophagy has emerged as a potential molecular bridge linking neural activity to tumor behavior. Modulating autophagy provides an additional avenue for therapeutic intervention, as demonstrated by pharmacological agents such as chloroquine and memantine. Translating these mechanisms into real therapies demands more precision. Because both neurotransmitter release and autophagy status between tumor-suppressive and supportive states remain vague. Conditionally activated prodrugs or targeted delivery platforms should be considered and constructed, therefore selectively restricting pathological neuro-tumor communication and keeping the autophagic baseline that normal neurons require. The physical architecture of these circuits, especially the feedback loops that connect the central nervous system to peripheral ganglia, remains largely unexplored. Before oncologists can integrate neural-targeted regimens into standard care protocols, large-scale clinical trials must strictly quantify the off-target neurological damage these agents inflict.

In conclusion, targeting the neural-tumor axis represents a promising therapeutic strategy in the field of oncology. Further elucidation of underlying mechanisms and more precise pharmacological interventions is required in the future. This review outlines the current understanding of neural–tumor interactions, identifies key signaling pathways with therapeutic potential, and suggests autophagy as a pivotal mechanism connecting the neural, tumor, and immune microenvironments. These findings provide valuable directions for future investigations and contribute to the emerging discipline of cancer neuroscience.

## Figures and Tables

**Table 1:** The key signaling pathways in neuro-tumor interactions

Pathway/Target	Classification	Mechanism	Disease	Ref.
Glutamatergic signaling pathway-AMPA/NMDA	synaptic communication	Neurons form functional bona fide chemical synapses with glioma cells or “pseudo-tripartite” structures with B2BM cell, and release glutamate, glioma/B2BM cells receive excitatory neural signals through AMPA/ NMDA receptors that promote tumor proliferation and invasion	Glioma, Breast cancer	60,64
	Paracrine signaling	1. Para-neuronal secretion of glutamate into the tumor microenvironment promotes tumor proliferation and invasion 2. Gliomas secrete massive excitatory neurotransmitter glutamate, which over-activates AMPA and NMDA receptors in neuronal cells, causing seizure disorders and leading to neuronal death	Glioma, PDAC	60
GABAergic signaling pathways-GABAA	synaptic communication	In neuron-glioma synapses, neurons produce GABA, depolarizing glioma cells, leading to elevated intracellular chloride ion concentrations and increasing glioma proliferation	Glioma	61
	Paracrine signaling	1. Para-neuronal secretion of GABA into the tumor microenvironment promotes tumor proliferation and invasion 2. Elevated glutamate concentration in the tumor microenvironment decreases KCC2 expression in peritumoral neurons, leading to GABA receptor activation of chloride efflux and functional excitation of neurons	Glioma, Breast cancer, Colorectal cancer	65
Cholinergic signaling pathways-CHRMs	synaptic communication	Neurons form cholinergic synapses with glioma, and glioblastoma receives acetylcholine signaling through CHRM3 receptor, promoting tumor proliferation and migration	Glioma	62
	Paracrine signaling	1. Enhancing Wnt and Notch signaling pathway activity to enhance tumor growth and metastasis through CHRM3 2.Inhibiting downstream MAPK/EGFR and PI3K/AKT pathways in PDAC cells through CHRM1	Prostate cancer, Gastric cancer, Pancreatic cancer	87,88
	Immunity modulation	Suppressing CD8+ cell infiltration and decreasing the Th1/Th2 ratio, leading to a suppressive immune microenvironment	PDAC	125
Adrenergic signaling pathway	Paracrine signaling	Sympathetic nerves release catecholamines, promoting tumor-cell proliferation, invasion, migration, angiogenesis, and resistance to cell death	Prostate cancer, Gastric cancer, Pancreatic cancer, Breast cancer, NSCLC, Colorectal cancer	83
	Immunity modulation	1. Agonizing astrocytes β2-AR, regulating TNFα and downstream inflammatory genes 2. Activating T cell β2-AR, leading to its depletion, impairing anti-tumor immune response 3. Activating MDSCβ2-AR, thus activating STATs signaling to regulate the expression of immunosuppressive factors and altering the proliferative capacity of T cells; reprogramming MDSC metabolism, leading to immunosuppression 4. Inhibiting cytotoxicity and cytokine production by NK cells	Colorectal cancer, Melanoma	111,124
Dopamine signaling pathway	Paracrine signaling	Activating dopamine receptor D2 on tumor cells to inhibit tumor growth	Glioma, Breast cancer, Lung cancer, Pancreatic cancer	224
	Immunity modulation	1. Antagonizing dopamine receptor D2, affecting the immune metabolism of TAMs and leading to a pro-inflammatory tumor microenvironment 2. Activating NK cell via D1-like receptors or inhibiting it by D2-like receptors	Glioma	108
MENK	Paracrine signaling	MENK binds opioid receptors on tumor cells and relocates NFAT1 to the nucleus, ultimately leading to apoptosis	Glioma, Melanoma, Colorectal cancer, Ovarian cancer	74
	Immunity modulation	Remodeling the tumor immune microenvironment, increasing the infiltration of macrophages, NK cells, CD8+ T cells, CD4+ T cells, and other immune cells, which is conducive to overcoming tumor immune escape and immune resistance	NSCLC, Colon cancer	130,131
BDNF-TrkB	synaptic communication	Promotes NGS formation and the transport of AMPA receptors on the surface of glioma cells, thereby amplifying glutamate-induced currents in malignant cells and promoting glioma growth	Glioma	75
	Paracrine signaling	Neurons or tumors secrete BNDF, binding to TrkB receptors to activate downstream signaling pathways and promote tumor proliferation and invasion	Breast cancer, Retinoblastoma, Oral squamous cell carcinoma, Melanoma	225
	Immunity modulation	1. Reducing microglia/macrophage infiltration and CD68 immunoreactivity, which in turn reduces glioma migration 2. Stimulating IL-15 production by microglia, shifting myeloid cells to an antitumor state 3. Re-educating TAM, developing an immunosuppressive microenvironment	Glioma, Prostate cancer, Lung cancer	109,110
NLGN3	synaptic communication	Promoting neuron-glioma synapse formation	Glioma	77
	Paracrine signaling	Activating PI3K-mTOR pathway activity in glioma cells and driving their proliferation, and inducing cancer stem cell characterization through autocrine	Glioma, Neuroblastoma	226
IGF1	Paracrine signaling	TuAstrocytes and olfactory receptor neurons indirectly or directly secrete IGF1 to promote tumor proliferation and progression	Glioma	80
LCN2	Paracrine signaling	Promoting tumor progression via STAT3 pathway	Medulloblastoma	81
Reelin	Paracrine signaling	SCLC secretes reelin to recruit reactive astrocytes, the latter promoting SCLC growth by secreting neuronal pro-survival factors	Brain metastases from SCLC	82
GDNF- GFRα1	Paracrine signaling	1. Nerve-released GFRα1 enhances tumor nerve invasion via GDNF-RET signaling 2. Endoneurial macrophages secrete GDNF, activating RET to induce PNI	PDAC	126
ARTN	Paracrine signaling	ARTN promotes pancreatic cancer invasion and perineural dissemination	Pancreatic cancer	92
NCAM1	Paracrine signaling	Direct contact between Schwann cells and cancer cells induces process formation and directed migration, a process that depends on NCAM1 in Schwann cells	Pancreatic cancer	93
NGF- TrkA	Paracrine signaling	1. Tumor secretes NGF, recruiting nerves into the tumor microenvironment and hijacking nociceptive nerves to secrete CGRP to support tumor growth 2. Nerves secrete NGF, inducing the tumor to migrate along nerves 3. Binding to TrkA receptors on the tumor, activating the PI3K/AKT and MAPK signaling pathways to promote tumor survival, migration, and invasion	Pancreatic cancer, Gastric cancer, Oral mucosa carcinoma	95
	Immunity modulation	Desensitizing the tumor to IFN-γ signaling, repelling T cells and NK cells, and suppressing T cell function	Melanoma	127
substance P	Paracrine signaling	Neurons secrete SP, inducing tumor cell death and ssRNAs release, indirectly activating tumor metastasis expression	Breast cancer	102
Midkine-CCL4-CCL5	Immunity modulation	Neurons secrete midkine, activating CD8+ T cells to produce CCL4, which in turn induces microglia/macrophages to produce CCL5, thus regulating LGG stem cell survival	Glioma	107
IL-6-STAT3	Immunity modulation	Astrocytes secrete IL-6, which binds to IL-6R and activates STAT3 signaling, thereby promoting tumor-cell proliferation and migration	Glioma	112
NAA/NAT8L	Immunity modulation	Neurons secrete NAA, inhibiting NK cells and CD8+ T cells, promoting immune escape from brain cancer cells	Breast cancer	114
CGRP	Immunity modulation	Nociceptor neurons in sensory nerves secrete CGRP, weakening anti-tumor immunity by accelerating CD8+ T cell depletion	Melanoma	116
TSP-1	synaptic communication	Glioma cells secrete TSP-1, promoting the synaptic connectivity of neurons and increasing glioblastoma proliferation	Glioma	63

**Table 2:** The promising drugs targeting neuro-tumor interactions

Classification	Drug	Target	Mechanism	Disease	Phase	Ref.
Neurotransmitter blocker	Talampanel perampanel	AMPA receptor	Blocking AMPAR, inhibiting glioma-induced epilepsy, and suppressing calcium-related invasiveness and growth of gliomas	Glioblastoma	II	60,206 NCT00064363
	Memantine	NMDA receptor	Blocking NMDAR, attenuating brain necrosis from radiotherapy, altering the immunosuppressive activity of TAM, and promoting anti-tumor immunity	Brain tumors, Hepatocellular sarcoma	III	205 NCT00566852
	Propranolol carvedilol	Adrenergic receptor	Blocking β-adrenergic receptor signaling, inhibiting the tumor from receiving catecholamine stimulation from multiple sources, and inhibiting tumor progression by reducing tumor cell proliferation, metastasis, modulating the immunosuppressive environment, and inducing death	Colorectal cancer, Prostate cancer, Breast cancer, Ovarian cancer, Glioblastoma, PDAC, Melanoma	IV	204 NCT02962947
	ONC201	Dopamine receptor	Blocking dopamine receptors, inhibiting proliferation, mediating apoptosis, and remodeling the pro-inflammatory tumor environment	Glioma, Prostate cancer, Endometrial cancer, Neuroendocrine tumors	III	108,209 NCT05580562
	Atropine	Choline receptor	Blocking cholinergic receptors, inhibiting tumor EMT, deregulating the immunosuppressive environment, and suppressing cancer stem cells	Colorectal cancer, Breast cancer, PDAC	IV	88,207,208 NCT00575523
Neurotrophin inhibitor	Tanezumab	NGF	NGF Antibody, relieving pain, inhibiting tumor invasion and migration, and reducing nerve infiltration	Cancer bone metastases, Pancreatic cancer	III	210 NCT02609828
	Larotrectinib Entrectinib	TRK	Inhibiting NTRK1/2/3, suppressing the signaling pathway associated with the aberrant proliferation of tumor cells due to persistent activation of the aberrant conformation of TRK proteins	TRK fusion-positive solid tumors	FDA-approved	211
Synapse inhibitor	Meclofenamate	Gap junctions	Inhibiting TM-dependent tumor intercellular communication, increasing susceptibility to chemotherapy	Glioblastoma	II	212 NCT02429570
	INCB7839	NLGN3-ADAM10	Inhibiting ADAM10, blocking NLGN3 shedding, and preventing tumor proliferation and progression	Glioma	II	77
Neuro-anesthetic	Botulinum toxin	Intratumoral innervation	Inhibiting acetylcholine vesicle release at the neuromuscular junction, suppressing tumor cells' proliferation, and promoting apoptosis	Head Neck Cancer, Gastric cancer, Breast Cancer, Neuroma, Cutaneous Leiomyomas	IV	NCT01374191
Other repurposing drugs	Aprepitant	Substance P	Inhibiting TACR1 and blocking ssRNA release from SP-activated dying cells, suppressing breast cancer cell growth, invasion, and metastasis	Breast cancer	Pre-clinical	102
	Telmisartan	IL-6	Agonizing PPARγ, inhibiting astrocyte IL-6 paracrine secretion, and inhibiting glial cell proliferation and migration	Glioma	Pre-clinical	112
	Rimegepant	CGRP	Blocking CGRP, inhibiting cancer cells from utilizing sensory nerves to induce protective autophagy	Oral mucosa carcinomas	Pre-clinical	96
	Fluoxetine	Glutamatergic synaptic transmission	Blocking NMDA receptors and interfering with glutamatergic synaptic transmission between neurons and glioma cells, exhibiting antiproliferative, pro-apoptotic, and synergistic chemotherapeutic anti-tumor effects	Glioma, Colorectal cancer	I	NCT05634707
	Imipramine	Serotonin	Blocking serotonin reuptake, inhibiting cell migration and invasion, inducing apoptosis, and augmenting autophagy, reprogramming of the immunosuppressive tumor microenvironment through inhibition of histamine receptor signaling	Glioma, breast cancer	I	33 NCT04863950
	Gabapentin	TSP-1	Pharmacologically inhibiting TSP-1, reducing glioma proliferation and network synchrony	Glioma	Pre-clinical	63

## Abbreviations

α-Syn: α-synuclein
BBB: blood-brain barrier
B2BM: breast-to-brain metastasis
β-AR: β-adrenoceptors
CAFs: cancer-associated fibroblasts
CGRP: calcitonin gene-related peptide
CNS: central nervous system
CMA: chaperone-mediated autophagy
CRC: colorectal cancer
DMG: diffuse midline gliomas
DRD5: dopamine receptor D5
GDNF: glial cell line-derived neurotrophic factor
GAP43: growth-associated protein 43
IGF1: insulin-like growth factor 1
IL-4: interleukin-4
LCN2: lipocalin-2
LGGs: low-grade gliomas
MMPs: matrix metalloproteinases
MENK: methionine enkephalin
MDSCs: myeloid-derived suppressor cells
NAA: N-acetylaspartate
NGF: nerve growth factor
NPCs: neural precursor cells
NLGN3: neuroligin-3
NGSs: neuro-glioma synapses
NMDARs: N-methyl-D-aspartate receptors
NSCLC: non-small cell lung cancer
OPCs: oligodendrocyte precursor cells
PDAC: pancreatic ductal adenocarcinoma
PNI: perineural invasion
PNS: peripheral nervous system
RIBI: radiation-induced brain injury
Tregs: regulatory T cells
SCLC: small cell lung cancer
SP: substance P
TMZ: temozolomide
TSP-1: thrombospondin-1
TME: tumor microenvironment
TMs: tumor microtubes
TNTs: tunneling nanotubes
TTYH1: tweety-homolog 1
VEGF: vascular endothelial growth factor
